# RETRACTION: Effect of Mitomycin C and 5‐Fluorouracil on Wound Healing in Patients Undergoing Glaucoma Surgery: A Meta‐Analysis

**DOI:** 10.1111/iwj.70362

**Published:** 2025-03-17

## Abstract

**RETRACTION:** CuiS. , ZhangJ. , ZhangS. , LiJ. , and LiQ. , “Effect of Mitomycin C and 5‐Fluorouracil on Wound Healing in Patients Undergoing Glaucoma Surgery: A Meta‐Analysis,” International Wound Journal 21, no. 3 (2024): e14500, 10.1111/iwj.14500.37990074 PMC10898392

The above article, published online on 21 November 2023, in Wiley Online Library (http://onlinelibrary.wiley.com/), has been retracted by agreement between the journal Editor in Chief, Professor Keith Harding; and John Wiley & Sons Ltd. Following an investigation by the publisher, all parties have concluded that this article was accepted solely on the basis of a compromised peer review process. The editors have therefore decided to retract the article. The authors did not respond to our notice regarding the retraction.

**RETRACTION:** S. Cui , J. Zhang , S. Zhang , J. Li , and Q. Li , “Effect of Mitomycin C and 5‐Fluorouracil on Wound Healing in Patients Undergoing Glaucoma Surgery: A Meta‐Analysis,” International Wound Journal 21, no. 3 (2024): e14500, 10.1111/iwj.14500.37990074 PMC10898392

The above article, published online on 21 November 2023, in Wiley Online Library (http://onlinelibrary.wiley.com/), has been retracted by agreement between the journal Editor in Chief, Professor Keith Harding; and John Wiley & Sons Ltd. Following an investigation by the publisher, all parties have concluded that this article was accepted solely on the basis of a compromised peer review process. The editors have therefore decided to retract the article. The authors did not respond to our notice regarding the retraction.

